# Frontal EEG/ERP correlates of attentional processes, cortisol and motivational states in adolescents from lower and higher socioeconomic status

**DOI:** 10.3389/fnhum.2012.00306

**Published:** 2012-11-16

**Authors:** Amedeo D'Angiulli, Joanne Weinberg, Tim F. Oberlander, Ruth E. Grunau, Clyde Hertzman, Stefania Maggi

**Affiliations:** ^1^Department of Neuroscience, Carleton UniversityOttawa, ON, Canada; ^2^Institute of Interdisciplinary Studies, Carleton UniversityOttawa, ON, Canada; ^3^Department of Cellular and Physiological Sciences, University of British ColumbiaVancouver, BC, Canada; ^4^Developmental Neurosciences and Child Health, Child and Family Research Institute VancouverBC, Canada; ^5^Pediatrics Department, University of British ColumbiaVancouver, BC, Canada; ^6^Department of Epidemiology and Health CareVancouver, BC, Canada; ^7^Department of Psychology, Carleton UniversityOttawa, ON, Canada

**Keywords:** socioeconomic status, event-related potentials (ERPs), EEG power, EEG asymmetry, auditory selective attention, salivary cortisol, executive control and self-regulation

## Abstract

Event-related potentials (ERPs) and other electroencephalographic (EEG) evidence show that frontal brain areas of higher and lower socioeconomic status (SES) children are recruited differently during selective attention tasks. We assessed whether multiple variables related to self-regulation (perceived mental effort) emotional states (e.g., anxiety, stress, etc.) and motivational states (e.g., boredom, engagement, etc.) may co-occur or interact with frontal attentional processing probed in two matched-samples of fourteen lower-SES and higher-SES adolescents. ERP and EEG activation were measured during a task probing selective attention to sequences of tones. Pre- and post-task salivary cortisol and self-reported emotional states were also measured. At similar behavioural performance level, the higher-SES group showed a greater ERP differentiation between attended (relevant) and unattended (irrelevant) tones than the lower-SES group. EEG power analysis revealed a cross-over interaction, specifically, lower-SES adolescents showed significantly higher theta power when ignoring rather than attending to tones, whereas, higher-SES adolescents showed the opposite pattern. Significant theta asymmetry differences were also found at midfrontal electrodes indicating left hypo-activity in lower-SES adolescents. The attended vs. unattended difference in right midfrontal theta increased with individual SES rank, and (independently from SES) with lower cortisol task reactivity and higher boredom. Results suggest lower-SES children used additional compensatory resources to monitor/control response inhibition to distracters, perceiving also more mental effort, as compared to higher-SES counterparts. Nevertheless, stress, boredom and other task-related perceived states were unrelated to SES. Ruling out presumed confounds, this study confirms the midfrontal mechanisms responsible for the SES effects on selective attention reported previously and here reflect genuine cognitive differences.

## Introduction

Developmental studies focusing on behavioural or imaging methods such as event-related potentials (ERPs) and other electroencephalographic (EEG) techniques have shown that frontal brain areas of children with higher and lower family socioeconomic status (SES) seem to be recruited differently during laboratory tasks probing executive attention and cognitive control (see reviews in Hackman and Farah, 2009; Lipina and Colombo, 2009; Raizada and Kishiyama, 2010). In particular, an increasing body of work is focusing on the relationship between SES and the neural responses underlying children's selective attention, especially ERP signatures occurring very early after stimulus presentation (e.g., 100–200 ms). The findings in a small number of studies on auditory selective attention focusing on some of those specific waveforms (D'Angiulli et al., 2008a; Stevens et al., 2009) suggest that, while higher SES children of various ages “filter out” distracters, lower SES children attend to distracters (the irrelevant information) as much as they attend to targets (the relevant information), without apparent differences in terms of behavioural performance. Furthermore, studies based on visual novelty paradigms have shown evidence of *frontal hypo-activity* in lower-SES children as reflected by reduced early activation, compared to that recorded in higher-SES comparison children (Kishiyama et al., 2009).

Generally, the mentioned findings have been interpreted in terms of cognitive differences even though the spatial resolution of the measures used cannot rule out the possible contributions of emotional or motivational processes controlled by overlapping or adjacent frontal functional networks. Specifically, the anterior attention system (Posner and Rothbart, 2007, see also Lipina and Posner, 2012) is thought to control or direct attention and action by modulating cognitive and affective processing through two major functional subdivisions of the anterior cingulate cortex (ACC). The differing environments of lower- and higher-SES children may reflect an instance in which the anterior attention system may develop divergent ways of integrating cognitive and emotional aspects involved in adaptation and self-regulation (Davis et al., 2002; Blair, 2010). Differential childhood experiences may influence aspects of brain development associated with emotion regulation and social behavior, influencing the maturation of key brain areas, such as the prefrontal cortex (PFC), as well as their neural networks and interactions (Farah et al., 2006; Hackman and Farah, 2009). Thus, the hypothalamic-pituitary-adrenocortical (HPA) axis controlling cortisol regulation plays an important role at the intersection of these brain areas and the physiological and behavioural regulatory processes (Hackman et al., 2010). Plausibly, midline frontal areas involved in executive functions and top-down control of the autonomic nervous system through the HPA axis may be vulnerable to aversive experiences associated with SES (Shonkoff et al., 2009). For instance, increasing evidence indicates that lower-SES children show heightened activation of stress-responsive systems as reflected by elevated cortisol (Lupien et al., 2000, 2001). Particularly relevant for the present study, Tomarken et al. (2004) found evidence of left frontal hypo-activity in lower-SES 12–14-year-old adolescents by examining resting EEG power alpha frequency asymmetry; this is consistent with the adult and developmental findings of correlations between aversive emotional reactions, increased cortisol, and left EEG hypo-activity—to be specific, greater right, relative to left, frontal activation (Coan and Allen, 2004; see Kim and Bell, 2006, for a review specific to childhood).

The few developmental studies directly assessing aspects of neural activity related to the anterior attention system in lower SES children have emphasized the importance of theta oscillations. In a longitudinal study of resting EEGs, Otero and colleagues, (Otero, 1994, 1997; Otero et al., 2003) followed two cohorts of 22 lower- and higher-SES children from the age of 18–30 months to 5–6 years. Although differences in EEG power between the two groups declined with age, there were persistent differences in frontal theta and occipital/left temporal alpha bands. Similar differential prevalence in theta EEG tonic activity was found in another study in older under-privileged children (Harmony et al., 1988), suggesting evidence of significantly more theta than alpha power in lower-SES children. Previously, we reported evidence also suggesting that differences between SES groups in task-dependent frontal and midline theta activation could reflect differences in effortful regulation during selective attention deployment (D'Angiulli et al., 2008b). It is known that event-related increases in theta power are correlated with unspecific factors such as attentional demands, task difficulty, and cognitive load (Schacter, 1977; Gevins et al., 1997, 1998; see reviews by Sauseng et al., 2006, 2010). Unlike the resting activity, task-dependent theta power is typically inversely related to activity in the lower spectrum of the alpha band power (Klimesch, 1999), therefore, if defining patterns of activity such as hypo-activity/asymmetry were observed for theta, these may reflect perceived mental effort during attention. However, so far no study has verified the possible functional significance of patterns of theta oscillations for SES differences in relation to children's attention.

Given the complexity of the brain processes involved in selective attention tasks, issues still remain regarding the concomitant factors that may explain the observed differences between the SES groups. Few SES studies have assessed possible confounds related to emotional states (e.g., anxiety, nervousness, stress, etc.) and motivational states (e.g., boredom, engagement, etc.). EEG and cortisol measurements alone may not be sufficient to disambiguate the relative functional contributions of the different neural systems. Children's self-reports may add another measure enabling researchers to better discern subtle psychological states associated, on one hand, with the attentional anterior system as reflected by EEG/ERP and, on the other hand, with the HPA axis as reflected by cortisol.

Building on the existing literature and following up to our prior research, the goal of our study was to investigate whether the previously reported midfrontal neural correlates of attention were partly correlated with emotional and motivational variables associated with selective attention. To this end, we examined the pattern of relationships between the neural correlates, cortisol levels, and self-reported affective/motivational states among lower- and higher-SES adolescents. Specifically, we (1) tested differences in neural correlates of selectivity of auditory attention between lower- and higher-SES children; (2) examined patterns of salivary cortisol over a typical school day, including pre- and post-task attention task periods; and (3) established if the observed neural changes were associated with general or task-dependent HPA axis reactivity. As reviewed earlier, lower-SES children seem to attend similarly to both relevant and irrelevant information, therefore, we expected to confirm this tendency in the pattern of the present ERP and EEG power results in relation to the frontal midline scalp sites. Further, we hypothesized that lower SES children would require more effortful control than the higher-SES and might perceive the task more negatively, in particular, as more stressful or more boring. Hence, we expected lower-SES children to show a pattern of (a) relatively greater theta frontal asymmetry, (b) higher cortisol reactivity to the task, and (c) more aversive emotion/motivation self-reports than higher-SES children.

## Materials and methods

### Participants

Twenty-eight children, all Caucasian, with no hearing impairments, were recruited from two schools in distinctly different socioeconomic neighborhood contexts: one attended and populated predominantly by students with higher SES and the other predominantly by students with lower SES. Since there is only a limited knowledge base, specifically on SES and EEG, it appeared suitable to use the extreme groups approach to enhance the detectability of anticipated effects (see Preacher et al., 2005). Therefore, children were carefully selected to represent non-overlapping SES groups and matched on other relevant characteristics described below. The children were selected from two prospective cohorts in the context of an ongoing larger scale study mapping “neural socioeconomic gradients” in medium-sized urban and rural centers in Western Canada.

To recruit participants, an information package was distributed to all parents whose children attended grade 6 at the two schools. Parents signed a consent form and completed a brief questionnaire on demographic and socioeconomic information about their family including a clause to consent to an extensive follow-up involving saliva and EEG collection. Parents and children each signed consent to disclose school records and grades. Materials explaining what was involved were included in the package and presented at the school to teachers and parents during small information sessions. The recruitment process was carried out in the context of an overarching prospective cohort longitudinal study targeting children from all ages and grades. Thus, only general information about the present study was provided to our target families and children. Hypotheses and purposes of the study were only given (verbally to children and through a written take-home page to parents) at debriefing after the study but not at the recruitment stage. After screening for SES information and school records, the prospective participants were matched on age, gender, ethnicity, grades, health, and “computeracy” (ownership and use of internet and computers, including video gaming). Fifty families were then re-contacted by mail, of which 36 returned signed consents for the present study. Children were given $5 for their participation, and also received a book of stickers at the end of the study. Written parental informed consent and children's active assent was obtained according to a protocol approved by research ethics boards of all institutions involved.

The final sample of 28 was obtained after exclusion of six participants from the original sample of 36 children; two children (one with higher and one with lower SES) were excluded before running the experimental session as the pre-recruitment questionnaires disclosed paediatric diagnosis of Attention Deficit/Hyperactivity Disorder or Fetal Alcohol Syndrome. Data from another six children were discarded and not submitted to further analysis after preliminary diagnostic analysis: two children (with higher SES) had an insufficient number of artifact-free or artifact-corrected usable EEG data; three children (all with lower SES) did not meet the minimal required behavioural performance threshold; and one (higher-SES) child's salivary cortisol values were not in the reliably readable standard range.

Table 1 summarizes the characteristics of the two groups by parents' and teachers' indications and schools' administrative records, all of which indicated participants who were clinically healthy, typically-developing children with no history of medication or referral to disability assessment or services. In both SES groups, the median of the combined average grades in arithmetic, reading comprehension, and written composition was A− (i.e., ~ 90%), with no difference in their rank distributions (Mann–Whitney *U* = 73.0, *p* > 0.80). Accordingly, all children in the two groups met expectations on performance in the standard provincial exams assessing their levels of numeracy, reading comprehension, and writing skills (Foundation Skills Assessment, *FSA*, BC Ministry of Education, 2002a,b,c). Although we were not successful in matching children one-to-one within a narrow age range, the groups were reasonably comparable on gender, age, and grade point average (GPA).

**Table 1:** **Family, neighborhood and demographic characteristics of the two groups of children studied**.

	**Socioeconomic status**		
	**High**	**Low**	
N^*a*^	14	14	
Mean age (SD)	12.9 (1.1)	13.7 (1.2)	
Gender (% females)^*b*^	64	57	
Mean (SD) FSA numeracy percentile score	69.55 (6.39)	57.21 (18.06)	
Mean (SD) FSA reading percentile score	76.85 (9.87)	68.07 (13.15)	
Mean (SD) GPA (year report cards)	2.71 (0.05)	2.67 (0.16)	
**PARENTAL SES^*d*^**			
Mean of median household income^*c*^ (SD)	70,507.88 (15,369.58)	38,366.83^*^ (21,290.96)	
Mode of self-reported income range	> 90,000	< 30,000	
(%)	(64%)	(43%)	
% Single parents	7	36	
% Parent unemployment	0	43^*^	
			Max–Min
Mean (SD) occupation	8.29 (1.20)	3.00 (1.11)^*^	9–1
Mean (SD) education	5.86 (0.95)	2.93 (1.21)^*^	7–1
Residence rank	7.00	1.00	7–1
Mean (SD) total SES score	87.00 (6.76)	27.79 (6.08)^*^	
Mean (SD) rank^*d*^	47.16 (4.62)	11.46 (4.96)^*^	52.5–3
Composite parent social position class	II	IV	I–V
**NEIGHBORHOOD SES^*e*^**			
			(Max–Min rank: 11-1)
% (Rank) Physical vulnerability	3.6 (1)^↓^	12.4 (9)	
% (Rank) Social vulnerability	3.6 (2)^↓^	21.0 (10)^↑^	
% (Rank) Emotional vulnerability	6.1 (3)^↓^	21.0 (10)^↑^	
% (Rank) Language/cognitive vulnerability	4.0 (1)^↓^	26.7 (11)^↑^	
% (Rank) Communicative vulnerability	2.4 (1)^↓^	10.5 (9)	
% (Rank) Total vulnerable children^*f*^	9.6 (1)	43.8 (11)^↑^	

Note: Significance threshold for multiple comparisons was set at p < 0.005 following Bonferroni correction.

a After exclusion of 3 Higher-SES and 5 Lower-SES cases (see text for details).

b Comparison of aggregate ERP data between females and males within the same SES group did not yield significant differences (see text for details).

c Canadian Dollars (taken from Statistics Canada et al., 2001).

d Computed using a revised version of Hollingshead Four Factor Index of SES (Hollingshead, 1975; Bornstein et al., 2003).

e from Kershaw et al. (2005).

f Based on the cumulative number of children manifesting one or more types of EDI vulnerability.

* t_(26)_ > 3.98 P < 0.001, two-tailed.

↑ and

↓ indicate vulnerability above and below the population mean, respectively, for the study region (Z > 5.16, p < 0.0001, two tailed).

### SES measurement

Socioeconomic information about the parents was first obtained through the brief questionnaire included in the information recruitment package. Items included occupation of parents, their education and income, disposable income, money spent in children's after school activities, clothes and shoes, and money spent on computer/internet items and computeracy. Parents had the option to respond to a category format, where they were presented with a range of values, or to volunteer detailed information such as their specific annual income. Care was taken to include families that lived in single units with unique postal codes. In the second stage of determination of sociodemographic characteristics, children's age, gender, ethnicity, grades, and health were determined directly from the school district database. More exact family income, occupation, and education levels were obtained through linkage of unique postal codes as well as personal identifiers with census data from Statistics Canada et al. (2001). Part of the individual information (i.e., age, gender, ethnicity, and health status) was double checked at the beginning of the experiment through brief in-take verbal questions.

For each student, SES scores were computed using an adapted version of Hollingshead Four-Factor Index of Social Status (Hollingshead, 1975; Bornstein et al., 2003) that provided a composite index including residential area quality, as well as parents' income, occupation, and education. Initially, all SES indicator scores were transformed to ranks across individuals so as to equate the rank structure of the four-factor SES categories. Then, the Hollingshead's algorithm was used to weigh and aggregate the measures. Given the collinearity among SES indicators, the composite SES categories or ranks were used in all analysis.

The SES indices of the two groups of children are provided in Table 1 in raw, ranked, or aggregate forms, depending on the type of measure. The highest occupation of either parent was rated using the Hollingshead categories 9–1, ranging from “higher executives” to “laborers/menial workers.” On the composite SES scale (highest = I, lowest = V), the higher-SES parents ranked II (corresponding to college graduates and managers/professionals) whereas the lower-SES parents ranked IV (corresponding to high school graduates and skilled workers) with respective non-overlapping income distributions. The percentage of single parents was 36% in the lower-SES group versus 7% in the higher-SES group. The percentage of unemployed parents was 43% in the lower-SES group versus 0% in the higher-SES group. Parental occupation, education, and family income data were all within the 99% confidence interval of the means for the respective neighborhood data from the most recent available Census data (Statistics Canada et al., 2001). Therefore, our samples appeared to be representative of the populations of reference, that is, lower- and higher-SES children with no current differences in functional outcomes.

In addition to Hollingshead's residential ranking, quality of residential area (neighborhood) was also defined according to developmental vulnerability estimates taken from Kershaw et al. (2005). The percentage of vulnerable children in the lower-SES neighborhood was 43 vs. 7% in the higher-SES neighborhood. Among 11 geographically incorporated city neighborhoods (population ~ 65,000), the lower-SES neighborhood ranked 1^st^ (Note: corresponding to *maximum rank* = 11, indicated in ascending rank, a larger percentage) for vulnerability, whereas the higher-SES neighborhood ranked 11^th^ (Note: corresponding to *minimum rank* = 1, indicating in ascending rank, a smaller percentage); in this urban area, the school predominantly attended by lower-SES children was granted *inner-city* school status and as a result received government funding to support various basic intervention programs (e.g., lunch program).

To properly frame our SES data, it is important to briefly mention the socio-geographical context in which the study took place in terms of how the studied neighborhoods compared to, or represented, the reality of the entire province of British Columbia, adding that the present context is not uncommon in Canada. Although the lower-SES neighborhood targeted the lowest average family income and education level in the specific region, the incidence of vulnerabilities in any one aspect of development could be estimated as comparatively small, provincially. Therefore, from the lower-SES population considered, it would be more likely to draw by chance a child that would not show functional issues rather than the contrary.

### Design

**Figure 1:**
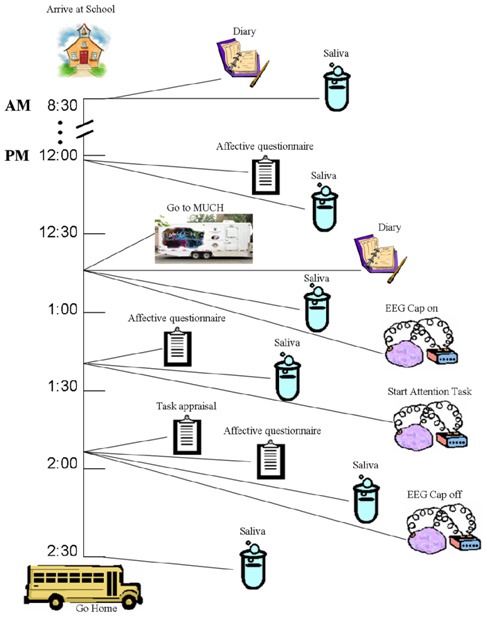
**Schematic representation of design and timeline of the study (see text for more details)**.

The experimental protocol is schematized in Figure 1. Children were seen individually across an entire ordinary school day. School days were chosen because children's daily routines (i.e., sleep, wake, and mealtimes) have been found to be much more regular than on weekend days (Davis et al., 1999). In addition, the school day appeared to provide the most ecologically valid, relatively controlled setting in which the ERP technique could be coordinated with repeated collection of saliva to evaluate cortisol patterns across the day as well as cortisol responses to the ERP task. To be able to better determine cortisol levels, and distinguish baseline vs. reactivity or recovery after the attention task as well as the global trend within the period of the school day, saliva was collected six times, four times before and two times after the ERP experiment. This choice was informed by the findings of the MacArthur Research Network on Socioeconomic Status and Health (2000). To exclude possible confounds in the cortisol data (food intake, sleep patterns, and intense physical activity), children completed a diary as soon as they arrived at school and after lunch noting when they awoke, type and timing of meals, level and type of physical activity, use of medications and general health during the week in which the study was being conducted.

The EEG recordings during attention testing and three of the saliva collections were conducted in a sound-proof, shielded EEG mobile lab (MUCH, Mobile Unit for Child Health, see D'Angiulli et al., 2005). The other three saliva collections were conducted in a quiet room in each school. The child was escorted in and out of his/her classrooms by one research assistant, to go to the mobile unit and to the quiet room. Pilot work using a virtually identical set-up showed that cortisol samples collected in the mobile lab were comparable to those collected in a quiet room within the school (Nordstokke et al., 2006; Oberlander et al., 2006).

### EEG/ERP data collection

#### Stimuli

Before the experiment we ensured that each child had pure-tone thresholds less than or equal to 20 dB HL in the range of 250–8000 Hz in both ears, using a portable Maico Diagnostics air conduction audiometer, model MA 27 (William Demant Holdings, Berlin, Germany).

**Figure 2:**
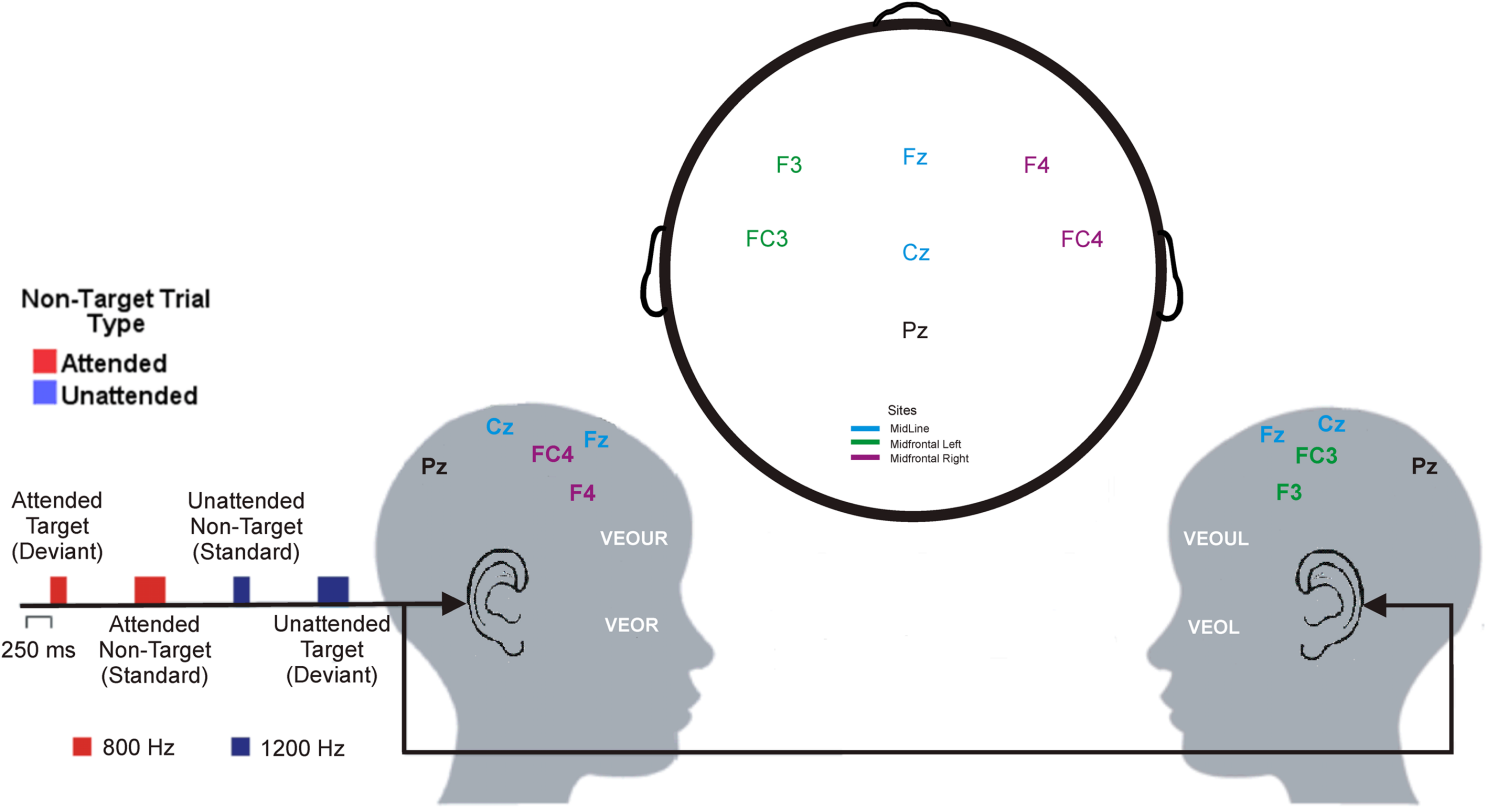
**Layout of the auditory selective attention task and electrode positions (adapted from the international 10–20 system of electrode placement) shown from the right side (left picture) and the left side (right picture) of a child's head.** As an example, this figure represents a child asked to press a button to the 800-Hz, 250 ms tone (*target* tone). Thus, the *attended* tone was 800-Hz, 100 ms tone (red) and the *unattended* tone was the 1200-Hz, 100 ms tone (blue).

The experimental stimuli were four pure tones, two frequencies (800 Hz and 1200 Hz) by two durations (100 ms and 250 ms) generated through STIM^2^ sound editor function program from Compumedics Neuroscan (Charlotte, NC, USA). Each stimulus tone was framed within a Hanning window of 250 ms with 10% (Rise/Fall of 5 ms) tapered at beginning and end of the tone. The level of the attenuation for both left and right channels were set below 90 dB. EEG was recorded during two blocks (either for 8- or 12-kHz tones) each consisting of 30 (10%) *target-duration tones* (either 100 or 250 ms), 30 (10%) *unattended target-duration tones* with the same duration as the target-duration tones but not the same frequency, 120 (40%) *attended non-target duration tones* with the same frequency as the target tones but not the same duration, and 120 (40%) *unattended non-target duration tones* (distracters) with a different frequency and duration from the target tones (see Figure 2).

The four tones were presented binaurally through insert earphones at 84 dB SPL, with an inter-stimulus interval of 1 s. The delivery of the tones was controlled via a Compumedics Audio System interfaced with the STIM^2^ program. Stimulus presentation followed different random orders for each block of trials and for each child; the different orders were randomly assigned to a given block and child, except that they were pre-selected so that a target tone would not appear immediately after the next target tone in the presentation sequence. The child was asked to press a button as fast and as accurately as possible to one of the tones (i.e., target), which was designated at the beginning of one of the two recording blocks. Reaction times and accuracy were measured from thumb press on a single button situated in the center of a hand-held response pad.

#### Data acquisition and recording procedures

The EEG was recorded using EEG Quik caps with silver chloride electrodes (Compumedics Neuroscan., Charlotte, NC, USA). Each participant had seven Ag-AgCl electrode sites (F3, F4, Fz, FC3, FC4, Cz, and Pz) applied according to the 10–20 system of electrode application (Harner and Sannit, 1974) as used previously (e.g., D'Angiulli et al., 2008a), and participated in a modified version of a standard selective attention task (Hillyard et al., 1973; see Figure 2). All electrodes were average referenced. Impedances were kept below 5 kOhms. The vertical electrooculograms (VEOG) were recorded from two split bipolar electrodes on the left and right supraorbital ridge (VEOGU, L, and R) as well as the left and right zygomatic arch (VEOG, L, and R). The signal from the electrodes was amplified and digitized by a SynAmps2 and a SCAN™ 4.3 EEG system (Compumedics Neuroscan, Charlotte, NC, USA) with filter settings at 0.15 Hz (high pass) and 100 Hz (low pass). The data from all channels were digitized online at a sampling rate of 1000 Hz.

#### EEG data reduction

Ocular artifact reduction was based on the eye movement reduction algorithm devised by Semlitsch et al. (1986). This algorithm consists of constructing an average artifact response and then subtracting it from the EEG channels on a sweep-by-sweep, point-by-point basis. To verify, confirm reliability, and validate our procedure, we correlated our edited data to the data obtained with two additional independently conducted procedures: a manual eye-movement rejection based on visual-score scanning procedure, and the eye-movement correction included in the EEGlab package (Delorme and Makeig, 2004). The agreement between the edited data with our procedure and the two confirmatory procedures was high (*r* = 0.87 with artifact rejection and *r* = 0.97, both *p* < 0.0001).

Initially, we analyzed 5-min resting eyes open/closed EEGs before and after the task, specifically focusing on the alpha band. Since the latter data was explained by individual differences seemingly unrelated to SES or any other dependent measure, we chose not to pursue the analysis of resting EEG further for this paper. However, consistent with our previous research (D'Angiulli et al., 2008a,b), we conducted a spectral power analysis of the single-trial EEG recordings. The focus of the present analysis was on age-appropriate theta and lower alpha (1 and 2) activation concurrent with Nd (encompassing a latency region between 200 and 500 ms), since both are known to vary according to attention, oddball, and working memory operations (e.g., Sauseng et al., 2006, 2010). To determine age-appropriate theta and lower alpha, individual alpha frequency band peaks were first identified and the cut-off point of lower alpha adjusted accordingly in each participant. In this way, theta bands ranged between 3.7 and 6.4 Hz, whereas lower alpha ranged between 6.5 and 9.5 Hz.

The EEG power peak was determined at the median of the waveform distribution corresponding to averages from non-target attended and unattended single trials, which in all frontal electrodes approximated 350 ms, i.e., the common “centering” for all the computed peaks. The extracted peak of the theta band frequency was expressed as percentage change above or below baseline level. *Attentional activation* was operationally defined as reflected by the *difference in theta band power between attended and unattended trials, in non-target (“standard”) stimuli*, not requiring response.

EEG band-specific frontal asymmetry continuous values were calculated using the peak power in the theta and lower alpha frequency bands from the averaged attended or unattended single trials in the non-target conditions. Symmetry values were calculated by taking the difference between the natural log of the total peak power recorded from midfrontal left electrodes minus the natural log of the total peak power recorded from midfrontal right electrodes [i.e., ln(F3 + FC3) − ln(F4 + FC4)]. Asymmetry scores for the unattended data could not be determined since a large proportion of the differences was zero. To obtain a better understanding of the relative distribution of left versus right asymmetry scores between the two SES groups, we also categorized the asymmetry values into two groups of scores. Negative asymmetry scores were categorized as showing relatively greater right hemispheric activation; whereas, positive asymmetry scores were categorized as showing relatively greater left hemispheric asymmetry. This categorization follows conventional assumptions in the literature (Coan and Allen, 2004).

#### ERP processing

Each participant's EEG was epoched (100 ms pre-stimulus and 900 ms post-stimulus) and averaged with respect to the onset of each tone. Averages were computed for both relevant (i.e., attended) and irrelevant (i.e., unattended) non-target tones, separately for 800 Hz and 1200 Hz. Analyses showed no significant differences as a function of type of pure tone, therefore, the ERPs were averaged across the two types of tones to yield relevant and irrelevant pure tone averages for each participant. Outliers were defined as EEG epochs exceeding ± 100 μV thresholds and eliminated through automatic artifact rejection. Baseline correction was based on the 100 ms pre-stimulus interval.

The effect of selective attention in the ERPs was operationalized by computing negative difference waveforms as in previous work in children of comparable ages (Loiselle et al., 1980; Berman and Friedman, 1995; Bartgis et al., 2003). ERP differences between *attended tones (same frequency but different duration compared to the target tone)* and *unattended tones (different frequency and duration compared to the target tone)* were calculated. (Note that these trials did not require manual responses). Amplitudes of the attention-related *difference negativity* (*Nd*) wave were calculated as the maximum negative deflection at the 200–500 ms interval in the ERP difference waveforms between attended and unattended target tones.

### Cortisol sampling

The daytime cortisol pattern was determined from saliva samples collected over the course of the school day, before (8:20 am, 12:15 pm, 12:45 pm, and 13:15 pm) and after (13:45 pm and 2:45 pm) the ERP session and completion of the attention task. To collect saliva, the child was asked to spit into a small plastic test tube. Saliva samples were stored at 4°C until sampling was completed. The samples were then brought to the laboratory, where they were stored at −20°C until assayed. All samples from any one participant were included in the same batch to eliminate within subject inter-assay variability. Samples were assayed by radioimmunoassay using the Salimetrics High Sensitivity Salivary Cortisol Enzyme Immunoassay Kit (Salimetrics LLC, Philadelphia, PA). All samples were assayed in duplicate, and duplicates varying by more than 20% were re-assayed. The inter-assay and intra-assay variation were 3.04 and 6.58%, respectively.

The first saliva sample was taken within a few minutes after arrival at school (8:20 am). Four samples were taken after lunch (lunch period was from 11:45 am to 12:30 pm, the day previous to the scheduled experiment time parents were reminded that children needed to avoid consuming food or drink that could interfere with cortisol, e.g., dairy products) at approximately 30 min intervals [12:15 pm (actual M = 12:16, SD = 0.04); 12:45 pm (actual M = 12:47, SD = 0.02); 13:15 pm (actual M = 13:17 SD = 0.01); 13:45 pm (actual M = 13:47, SD = 0.01)]. Of the post-lunch samples, the first two were obtained to evaluate cortisol changes due to the EEG capping procedure and the subjective experience of the experimental session, as reported in previous neuroimaging studies (e.g., Tessner et al., 2006). To determine cortisol responses pre- vs. post-ERP testing, the target samples were those collected at 13:15 pm, immediately before starting the attention task, and at 1:45 pm, immediately after completion of the task. The ERP session lasted 30 min. Saliva was also obtained before the children went home (14:45 pm) to measure possible differences between lower- and higher-SES groups in returning to baseline (recovery) after the ERP session, as well as to assess more fully the expected decrease in cortisol levels over the school day.

In addition to school day cortisol, we derived an index of *task reactivity* by calculating the percent of change in post-task cortisol levels as compared to baseline, namely as:

[(baseline level− post-task level/baseline level)×100]

As baseline cortisol level, we selected the second cortisol sample, collected in the school at 12:15 pm, 30 min before going to the mobile lab and starting the task. Thus, the task reactivity could be assumed as most likely reflecting the changes in children's cortisol occurring in relation to what happened specifically during our attention task, as opposed to *general reactivity* (i.e., participating in the experiment). To distinguish general reactivity from task reactivity we calculated a further index measuring the percent change in pre-task cortisol levels as compared to baseline.

[(baseline level−pre-task level/baseline level)×100]

We used the same baseline measure used to obtain the task reactivity index; however, as pre-task cortisol level, we selected the fourth cortisol sample, collected at the start of the attention task after children had spent time wearing the EEG cap.

Cortisol data were examined for outliers, defined as any value more than ±3 SD from the mean (Gunnar et al., 1989; Ramsay and Lewis, 2003). Two children had outlier values for cortisol. These values were “winsorized” following the method of Tukey (1997), which involved replacing the outlier value with the closest value within the 3 SD range, and then included in the data analyses.

### Emotional state and task appraisal questionnaires

Before and after the attention task, the children completed a five-point rating scale measuring multiple affective states, containing eight age-appropriate items adapted from a standard *affective questionnaire* (Usala and Hertzog, 1989) (see Appendix A), and a post-test *task appraisal questionnaire* (Tomaka et al., 1999) (see Appendix B). Both types of items were pre-selected from the much larger sets included in the original sources based on previous extensive pilot work (D'Angiulli et al., 2007). Through the task appraisal questionnaire, children self-rated their perceived levels of engagement, difficulty, stress, fear, and coping attributed to the attention task. The affective questionnaire was administered three times, one at 12:15 (pre-test 1) to control for anticipatory reactive states, immediately before the ERP session at 13:15 (pre-test 2), and at 13:45 (post-test) upon completion of the ERP session. The task appraisal questionnaire was administered immediately after the post-test affective questionnaire. The collection times are shown in relation to the entire design of the study in Figure 1.

### Analytic strategy

In all analyses, we used GLM through either ANOVAs, focused contrasts, or multiple regressions. Repeated measures ANOVA models used Greenhouse–Geisser adjustment. Bonferroni correction was used to adjust for multiple comparisons. Analyses were based on valid standard trials (i.e., correctly withheld responses). The rationale for our analyses strictly followed the predictions linked to the hypotheses put forward in the introduction.

Initially, we conducted preliminary analyses showing that differences between the SES groups were not associated with group confounds in accuracy or reaction times (given that the task had been pre-calibrated to keep all children at approximately the same performance level).

We then tested the prediction that the higher-SES group would show a greater ERP differentiation between attended (relevant) and unattended (irrelevant) tones than the lower-SES group. For consistency with previous results, all electrodes were analyzed individually to establish the effect sizes associated with the Nds for each electrode, split by SES group. Successively, the individual electrodes were included as separate levels in a 2 (Group: lower-SES and higher-SES) × 7 (Electrode placement: PZ, CZ, FC3, FC4, F3, FZ, and F4) ANOVA.

Following this preliminary ERP analysis, the different electrodes were aggregated (i.e., EEG signal was collapsed over electrodes by averaging) to reflect coarsely the main subdivision of the attentional networks (Posner and Rothbart, 2007). That is, the analysis focused specifically on four groups of electrodes: *parietal* (PZ), corresponding to the posterior attention system, *midline* (CZ and FZ), *midfrontal left* (F3 and FC3) and *midfrontal right* (F4 and FC4), corresponding to the main parts of the anterior attention system. Thus, to test the hypothesis that lower SES children attended and monitored irrelevant stimuli significantly more than their higher SES counterparts, over the two frequency bands, we ran a 2 (Frequency Band: theta vs. lower alpha) × 4 (Electrode Group) × 2 (SES Group) ANOVA with Attentional Activation Difference as the dependent variable.

Furthermore, we assessed whether:

1. Any event-related power asymmetry effects were present, to test the hypothesis of left hypo-activity during the attention task,
2. The two groups differed globally in cortisol when examined during their school day, including before and after the attention task,
3. Pre-selected self-rated emotional and motivational state items differed in the two groups before and after the task, and the two SES groups differed for emotional states associated with task appraisal (e.g., difficulty, stressfulness etc.)

All these analyses used simple or polynomial contrasts based on ANOVA models.

Based on the literature, we expected very large individual differences in cortisol changes capable of overshadowing group effects, especially given our modest sample size. Analysis of individuals in the groups is, in cases such as the present one, a very valuable tool to detect subtle differences in mechanisms that may have important functional implications for neurobiological processes (Kosslyn et al., 2002). Therefore, the final stage of our analysis focused on the hypothesis that individual variations in greater attentional activation changes in theta power for the right midfrontal electrodes would be associated with individual variations in SES rank, task-dependent HPA axis reactivity, and motivational changes measured at the beginning of the task (also expressed as percentage change from pre-task baseline) but not with individual variations in general HPA axis reactivity. This hypothesized pattern of relationships was assessed through a single multiple regression model in which the predictors were entered serially as separate blocks (in the same order as above) so we could assess the relative contribution of each variable.

## Results

### Behavioural

Reaction times, accuracies, and false alarms did not differ significantly between lower- and higher-SES children across different stimulus conditions (Table 2). The overall average performance accuracy was over 80% and false alarms were below 5%. This also reflected individual differences as the individual accuracy was over 75% and below 90%, indicating the attention task difficulty level was moderately easy, yet not at ceiling. Overall the groups displayed similar behavioural response levels which, therefore, cannot account the differences in the EEG/ERP patterns. Collapsed across groups, correlations between aggregate mean ERP amplitude and accuracy or RTs for hits and false alarms yielded small effects (0.08 ≤ *r* ≤ −0.17, *p*'*s* > 0.42).

**Table 2:** **Behavioural profiles (and statistical comparisons) of the two groups of children in relation to the auditory selective attention task (responses to deviant attended tones)**.

	**Socioeconomic status**			
	**High (*n* = 14)**	**Low (*n* = 14)**	***t*_(26)_**	***P***
**ACCURACY %**				
Hits	84.52 (11.92)	80.48 (19.45)	0.66	0.51
False alarms	3.38 (4.43)	4.88 (5.90)	−0.76	0.45
**REACTION TIME IN MS**				
Hits	569.84 (50.82)	571.64 (57.93)	−0.09	0.93
False alarms	506.72 (71.26)	501.38 (71.04)	−0.19	0.84

Note: Values represent group means (values in parentheses represent standard deviations) collapsed across tone frequency conditions which did not reveal significant differences on preliminary analyses. Accuracy is in percentage; reaction times are in milliseconds.

### EEG/ERP

#### Negative wave differences

**Figure 3:**
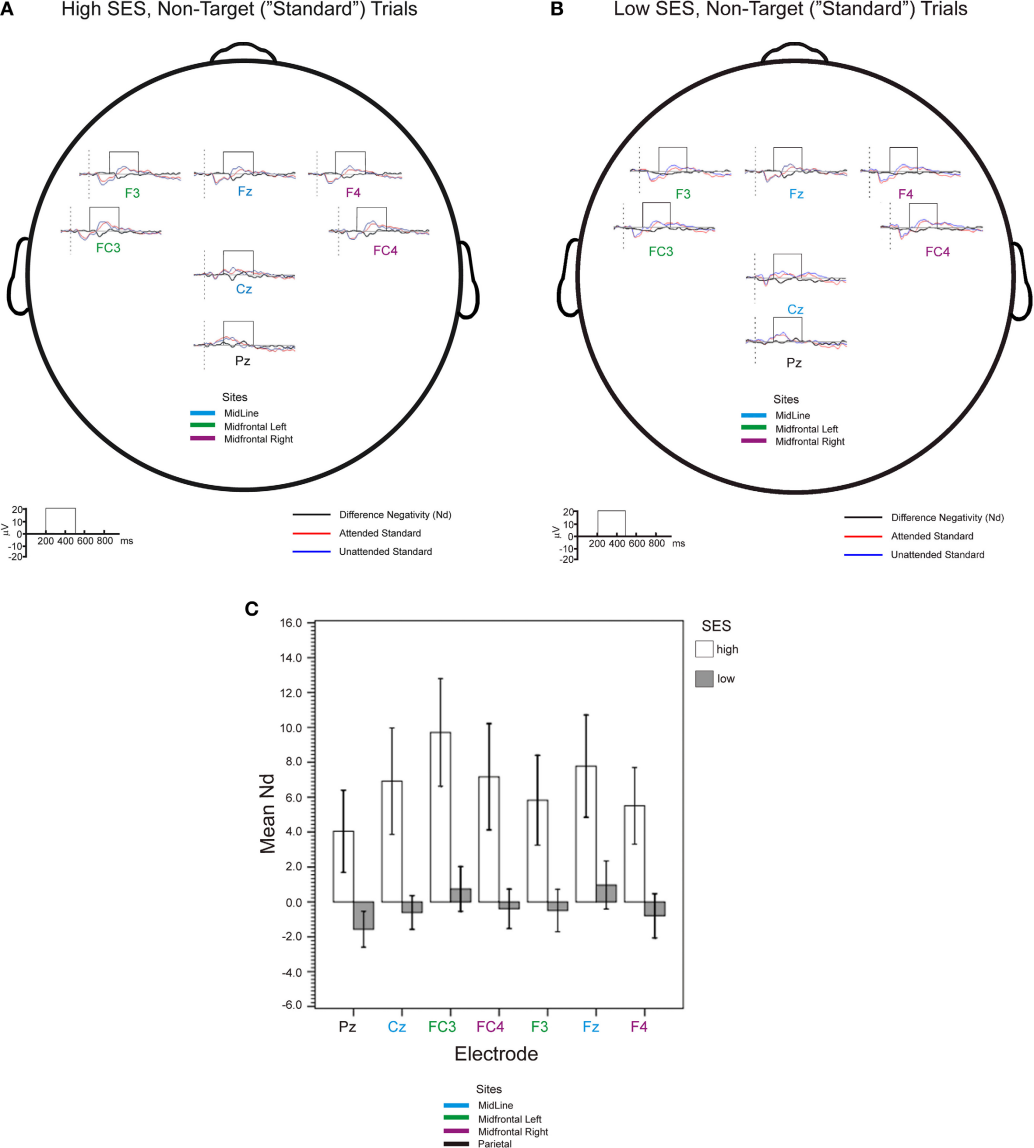
**Group-mean event-related potentials (0–30 Hz) for the high-SES (A) and low-SES (B) children, averaged with respect to attended (red) and unattended (blue) tones are shown for high and low SES children at all tested electrode sites.** Difference evoked waveforms (attended - unattended) are shown below the standard waveforms in black. **(C)** Central outcome of mean difference negativity (Nd) analysis (see windows in panels **A** and **B**); error bars represent ± 1 SE.

Figure 3A and 3B shows waveforms and Nd amplitudes for higher and lower-SES groups, respectively, for attended and unattended standard stimuli. Figure 3C shows the central outcome of the observed Nds. A mixed-model ANOVA, Electrode (7 levels) × SES Group (2 levels), revealed no interaction (*F* < 1) and an effect of SES Group [*F*_(1, 26)_ = 6.79, MSE = 354.98, *p* = 0.01, η^2^_*p*_ = 0.21] indicating that the Nd amplitudes were more negative for higher- than lower-SES children (*median of mean Nds*: 6.81 μV). However, there were also differences between electrodes [*F*_(1, 76)_ = 3.23, MSE = 30.85, *p* < 0.05, η^2^_*p*_ = 0.11]. A follow-up polynomial contrast showed that the pattern in Figure 3C is well described by a quadratic trend [*F*_(1, 26)_ = 6.83, MSE = 18.14, *p* = 0.01, η^2^_*p*_ = 0.21] indicating a larger Nd in the midfrontal electrodes. Since the pattern of results was consistent with aggregating the electrodes, to test more focused hypotheses about the anterior attention system, we ran an Electrode Group (parietal, midline, midfrontal left, midfrontal right) × SES Group ANOVA. The results for main effects were virtually identical to the analysis on all electrodes, but Bonferroni pairwise comparisons clearly confirmed that, although the Nds were of similar magnitude at midline and midfrontal left electrodes, they were also significantly (*p* < 0.05) larger than Nds at parietal and midfrontal right electrodes.

There were no significant differences associated with gender within either SES group or when the two SES groups were collapsed. Furthermore, there were no significant interactions between Gender and SES when the ANOVA model included Gender as another factor.

#### Event-related EEG band power differences

**Figure 4:**
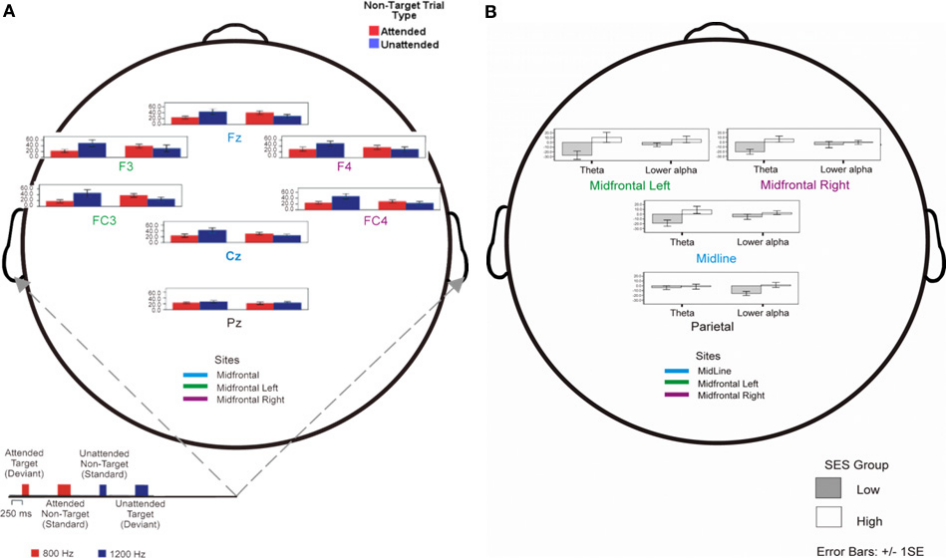
**(A)** Group-mean event-related theta (3.7–6.4 Hz) power of single trial data (non-target tones) averaged with respect to attended (red) and unattended (blue) tones are shown for high and low SES children. **(B)** Attended vs. unattended EEG Power peak percentage change over baseline in standard trials (attentional activation) for theta and lower alpha frequency bands.

We compared mean theta peaks for all electrodes in attended and unattended conditions in the two SES groups; the comparison is shown in Figure 4A. We found a three-way interaction between Group, Electrode Placement and Attention Condition [*F*_(1, 26)_ = 3.44, MSE = 235.79, *p* < 0.05, η^2^_*p*_ = 0.13] and a two-way interaction between Group and Attention Condition [*F*_(1, 26)_ = 7.36, MSE = 235.79, *p* < 0.05, η^2^_*p*_ = 0.25], as well as a main effect of Electrode Placement [*F*_(1, 26)_ = 2.93, MSE = 235.79, *p* < 0.05, η^2^_*p*_ = 0.12]. For all sites but PZ, there was a cross over interaction in which higher-SES children showed higher power level for attended than for unattended trials, whereas the lower-SES children showed the opposite pattern. Repeating the analysis without the PZ data, both three-way interaction and Electrode Placement effects vanished, confirming the crossover effect of Group by Attention Condition for all frontal sites [*F*_(1, 26)_ = 8.38, MSE = 218.23, *p* < 0.01, η^2^_*p*_ = 0.27]. Thus, other additional neural processes were associated with attending to irrelevant tones, in lower-SES children.

Figure 4B shows the difference in EEG power change in relation to attended vs. unattended trials (Attentional Activation) for theta and lower alpha bands over the aggregated electrodes in the two SES groups. Again, a three-way interaction Frequency Band × Aggregated Electrodes × SES Group ceased to be significant once the parietal site was excluded from the ANOVA model. A main effect of frequency band showed that theta showed ~21% more overall attentional activation than lower alpha [*F*_(1, 26)_ = 13.99, MSE = 1051.04, *p* < 0.001, η^2^_*p*_ = 0.40]. There was also an interaction between Frequency Band and SES Group [*F*_(1, 26)_ = 7.41, MSE = 1051.04, *p* < 0.05, η^2^_*p*_ = 0.26] and a main effect of SES Group [*F*_(1, 26)_ = 9.37, MSE = 562.39, *p* < 0.01, η^2^_*p*_ = 0.31]. This pattern of results could mainly be explained by the effect of SES Group significance for the theta [*F*_(1, 26)_ = 8.38; MSE = 1961.81, *p* < 0.01] but not for the lower alpha data (*F* < 1).

#### Frontal theta asymmetry and SES group

**Figure 5:**
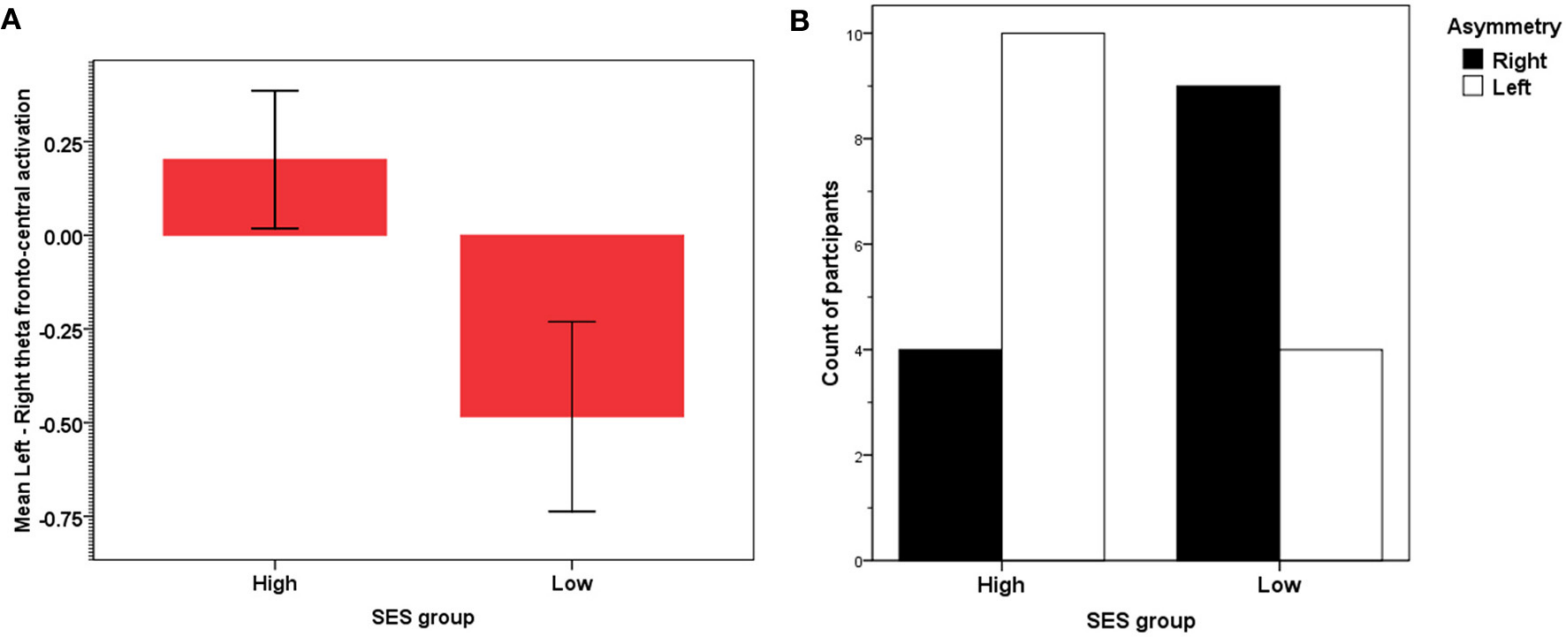
**(A)** Frontal asymmetry in theta activation in high- and low-SES children. **(B)** Relative frequency distribution of right vs. left frontal theta asymmetry in high- and low-SES children.

For the theta band, lower-SES children showed a marked right activation asymmetry whereas higher-SES showed the opposite pattern [*t*_(26)_ = 2.21, *p* < 0.05], see Figure 5A. As shown earlier (i.e., Figure 4B), lower alpha decreased globally as theta increased; because of this well-documented overall inverse relation, characteristic of event-related power [see review in Klimesch (1999)], we did not find any significant asymmetry for the lower alpha [*t*_(26)_ < 1]. When we compared the relative distribution of left vs. right asymmetry categorical scores between the two SES groups for theta, a significant difference was found between the two SES groups' distributions of participants showing left vs. right activation [χ^2^_(1)_ = 4.46, *p* < 0.05]. More individuals in the lower-SES group had relatively greater right than left activation (64 vs. 36%, *Z* = −2.18, *p* < 0.05), whereas in the higher-SES group, more individuals had relatively greater left than right (71 vs. 29%, *Z* = −3.46, *p* < 0.001) activation (see Figure 5B).

### Questionnaire data

In relation to self-rated emotional/motivational states, there were no significant changes from pre- to post-test except, importantly, for the *bored* item [*F*_(1, 26)_ = 9.03, MSE = 0.32, *p* < 0.01, η^2^_*p*_ = 0.27]. Children's self-reported boredom declined from 12:15 pm (Pre-test 1) to 13:15 pm (Pre-test 2) but returned to initial values at 13:45 pm (Post-test). There were however no differences between the two SES groups.

The task appraisal data showed no reliable differences between the SES groups. In addition, both SES groups reported significantly higher ratings for stress induced by the task as compared to stress as an internal affective state [mean task appraisal: 2.14 (SD = 1.24) vs. mean affective state: 1.36 (0.68); *F*_(1, 26)_ = 8.57, MSE = 1.01, *p* < 0.01, η^2^_*p*_ = 0.25], suggesting that the task was mildly stressful.

### Cortisol data

A mixed-design ANOVA, 2 (SES) × 6 (Collection Time for cortisol), was computed with repeated measures on Collection Time. There was a main effect of Collection Time [*F*_(2, 52)_ = 4.95, MSE = 0.19, *p* < 0.01, μ^2^_*p*_ = 0.19] and an effect of SES [*F*_(1, 25)_ = 3.57, MSE = 0.04, *p* < 0.05, μ^2^_*p*_ = 0.12], but no interaction effect (*F* < 1). Overall, cortisol levels in lower-SES children were marginally higher than levels in higher-SES children. However, the two groups displayed a similar pattern of cortisol secretion over the school day, with highest levels in the morning, and levels progressively declining over the day [*F*_(1, 25)_ = 5.60, MSE = 0.03, *p* < 0.05, μ^2^_*p*_= 0.18].

The index of task cortisol reactivity we used (see analytic strategy section) showed a mean change of 29.23 and 26.80% in the lower and higher SES group, respectively. In contrast, general reactivity showed a mean change of 0.93 and 26.80% in the lower and higher SES groups, respectively. For both groups and for both reactivity types the inter-individual variability was very large (in all cases, standard errors were between 22–23%). Thus, we could not detect any significant SES differences in reactivity. Although in the right direction as we expected, the differences between the groups were basically washed out by inter-individual variation, this means that given our very modest sample size, one way to detect genuine differences associated with SES was to use a regression approach focusing on individual differences effects. This was pursued in the next analysis.

### Right midfrontal theta power, SES rank, post-task cortisol reactivity, and boredom

We built a multiple regression model to test the hypothesis that individual variation in theta attentional activation at the midfrontal right electrodes would be predicted by individual variation in SES, changes in motivational state at the beginning of the task as reflected by self-rated boredom (the only questionnaire item which in the other analysis yielded reliable significant effects in relation to task time-course), and reactivity specific to our attention task, as opposed to individual variation in general reactivity. The results of this analysis are shown in Table 3. The gradient of midfrontal right attentional activation was associated with individual differences across SES rank, task reactivity, and increase in boredom from baseline to onset of the task. When general reactivity was included in the model, its effects were null, while including task reactivity significantly added explained variance to the model.

Table 3**Results of multiple regression models examining relationships between individual variation in theta attentional activation in midfrontal right sites and individual variation in cortisol reactivity and boredom increase in relation to the attention task**.

**Table:** 

				**Change statistics**				
**Model**	**R**	**R square**	**Adjusted R square**	**Std. error of the estimate**	**R square change**	**F change**	**df1**	**df2**	**Sig. F change**
**MODEL SUMMARY**									
1	0.532^*a*^	0.283	0.251	21.453	0.283	8.693	1	26	0.003
2	0.658^*b*^	0.432	0.378	19.540	0.149	5.518	1	25	0.013
3	0.737^*c*^	0.543	0.475	17.958	0.111	4.862	1	24	0.018
4	0.743^*d*^	0.552	0.458	18.247	0.009	0.372	1	23	0.534

a Predictors: (Constant), SES Rank.

b Predictors: (Constant), SES Rank, Task Reactivity.

c Predictors: (Constant), SES Rank, Task Reactivity, Boredom Change.

d Predictors: (Constant), SES Rank, Task Reactivity, Boredom Change, General Reactivity.

**Table:** 

	**Model**	**Unstandardized coefficients**		**Standardized coefficients**		**T**	**Sig.**
		**B**	**Std. Error**	**Beta**			
**COEFFICIENTS^*a*^**							
1	(Constant)	−28.475	9.121			−3.122	0.005
	SES rank	0.724	0.246	0.532		2.948	0.008
2	(Constant)	−27.086	8.329			−3.252	0.004
	SES rank	0.691	0.224	0.508		3.082	0.006
	Task reactivity	0.177	0.075	0.387		2.349	0.030
3	(Constant)	−27.714	7.660			−3.618	0.002
	SES rank	0.714	0.206	0.525		3.462	0.002
	Task reactivity	0.186	0.069	0.406		2.678	0.015
	Boredom change	0.139	0.063	0.334		2.205	0.039
4	(Constant)	−29.780	8.488			−3.509	0.002
	SES rank	0.740	0.214	0.544		3.461	0.003
	Task reactivity	0.254	0.131	0.554		1.929	0.065
	Boredom change	0.156	0.070	0.377		2.229	0.038
	General reactivity	−0.052	0.086	−0.180		−0.610	0.326

a Dependent variable: theta midfrontal right activation.

Correlations among SES rank, task reactivity, and boredom increase ranged from −0.06 to 0.06, which rules out multicollinearity artifacts.

## Discussion

This study examined the relationships among EEG correlates of attentional processes, salivary cortisol levels and emotional states in two groups of adolescent children representing the opposite ends of the SES spectrum. Consistent with previous results and some of our hypotheses, we found that the higher-SES group showed a greater ERP differentiation between attended (relevant) and unattended (irrelevant) distracters in midline electrodes. EEG power analysis showed that of those frequency bands analyzed, the most important results were found for theta. In particular, there was a crossover interaction between SES group and attention condition on theta peak power: lower-SES participants showed significantly higher power when ignoring tones rather than attending to them, whereas, higher-SES participants showed higher power when attending to tones rather than ignoring them. One possible interpretation is that, due to the fact that they live in less predictable and more threatening environments, lower-SES children may have learned the tendency to attenuate attentional selectivity, allocating relatively greater attention to distracters. However, to perform like their higher-SES counterparts they would also need to exert more effortful control. Thus, presumably the observed EEG/ERP pattern of results may reflect this background of differential processing “preference” (D'Angiulli et al., 2012) which the lower-SES children brought with them into the task.

EEG frontal asymmetries were also compared. In the attended channel, a significant difference between SES groups was found in the distribution of participants showing left vs. right frontal theta activation: This result is consistent with the finding of hypo-activity of the left frontal areas in lower-SES adolescents reported in another study Tomarken et al. (2004). The finding of left hypo-activity, however, in our case invites an interpretation that is quite different from the one proposed by Tomarken and colleagues (who linked hypo-activity to depression and psychopathology). First our effects were observed not on resting EEG but on event-related activity power; and second, those effects were observed in a sample of children with no known mental health problems or psychopathology or comorbidities, the children differed mostly on the environment in which they lived. Thus, our analysis revealed significant individual variations in the increase of selective attention in the two SES groups: Theta power difference increased with SES and involved right midfrontal electrodes. Since theta increased in the expected inverse fashion relative to lower alpha (Klimesch, 1999), our data are genuine evidence of event-related asymmetry, as theta asymmetry is exactly what would be expected when no reliable differences would be detectable for alpha (i.e., because of floor effects). Furthermore, we controlled for the most important confounds: motor requirements/response demands and task difficulty (see Andreassi, 2000; Cacioppo et al., 2000) by considering only standard (non-target) trials requiring no response, and counterbalancing the different tone features (frequency and duration). Thus, our results are not only reliable, but also novel, since to our knowledge this is the first study on event-related asymmetry focusing on SES influences in children. Given the link between attention deployment and subjective perception of mental effort (e.g., Pribram and McGuinness, 1977, 1991; Howells et al., 2010), the broader psychological/functional implication is that the frontal asymmetry differences observed in the theta band in lower SES children reflect level of perceived mental effort during the selective attention task.

We also found group differences in overall cortisol levels and an association between individual variation in EEG power and task-dependent HPA reactivity, associated with individual SES rank and an increase in boredom at the start of the task. However, SES did not predict either boredom or cortisol reactivity to task. In addition, the regression models indicated that SES remained a significant predictor of theta power even after controlling for boredom and reactivity, suggesting independent effects. These results do not seem to support the hypothesis that either perceived stress or boredom related to the task was confounded with frontal cognitive functions. Instead, our findings seems to suggest that SES effects were independent of task engagement or perceived stress and SES effects on frontal functions may be independent of these measures. One novel contribution of our study was to present data controlling for motivational aspects and perceived stress during a cognitive task, this type of data can better inform interpretation of frontal EEG or ERP results in SES research eliminating the possibility of confounds (Jolles and Crone, 2012). Indeed, our results confirm that it is unlikely that previous ERP/EEG findings concerning SES effects on selective attention could be attributed to confound due to the variables considered here.

Our cortisol results provide some evidence that the brain areas implicated are part of the anterior attention system. With the results from the power analysis, the finding of differential patterns of relationship between selective attention and cortisol reactivity suggests that lower-SES children may have used more executive resources to control for the processing of (and response inhibition to) irrelevant information than did the higher-SES children. Importantly, as noted, components of the anterior attentional system are believed to be involved in the regulation of reactive, emotion-related systems, such as the HPA axis (see Davis et al., 2002; Blair et al., 2005). From the point of view of developmental psychobiology, these differences in neural processes in lower- and higher-SES do not necessarily imply a behavioural performance gap, but may instead be interpreted as part of different coping or even motivational responses enabling children to adapt to environments which present different types of information-processing challenges. Given that lower- and higher-SES children live in very different environments, these two groups might develop experience-dependent patterns of neural activity and self-regulation that would be differentially and preferentially associated with selective variations in attention and executive processes to differentially match the types of environmental challenges they most frequently encounter (Blair, 2010). It would have to be seen whether and which functional consequences could be associated with SES influence in more difficult tasks than ours, in which overall behavioural performance is not above 80%. This is an important empirical question for future research.

The purpose of the present study was to isolate the effect of SES under the assumption [well supported by the literature, see review by D'Angiulli et al. (2012)] that SES is a proxy of social-environmental conditions that are known to influence development quite substantially and, therefore, to focus on one specific task that we hypothesized to be independent from prior physical and mental health conditions, academic achievement or cognitive outcomes. As shown by the broad literature on cognitive performance, school achievement, physical, and mental health, outcomes are often consequences of exposures to unfavorable environments which tend to correlate with lower SES. Without controlling for the contribution of those consequences from our analysis, it would not be possible to make inferences about the role of SES on the processes of interest. In other words, if there was variation in the sample in terms of physical and mental health, cognitive performance, and school achievement, we would be in no position to infer that the differences we found between the groups were indeed associated with SES environment rather than other factors such as health and cognitive skills. Consequently, the matched design we used is accepted as a rigorous method to account for the effect of known confounders (Jackson and Verberg, 2006). In addition, as the current sample is drawn from a larger study, the epidemiological background data of the geographical context in which our study was conducted (Kershaw et al., 2005) show that the lower-SES sampling distribution of reference is a variegated one in which drawing non-vulnerable cases should actually be more likely (specifically, *P* = 0.66) than drawing vulnerable ones. Hence, far from representing a form of bias, and given our scope, the sample matching we used insured “translation validity” (Trochim, 2000). Still, our sample-matched design leaves open the empirical question of what would the pattern of findings look like had we used the alternative design (i.e., unmatched SES samples).

In conclusion, we found ERP differences between lower- and higher-SES children *without* differences in their concurrent behavioural performance. EEG power analysis suggests that children from the two groups recruited different neural processes to obtain similar behavioural performance levels. Relative to higher-SES children, lower-SES children engaged resources to also attend to irrelevant auditory information. The individual differences relationship between SES, cortisol reactivity, and frontal activation suggests that lower-SES children used additional compensatory resources to monitor/control response inhibition to distracters, perceiving also more mental effort (reflected by theta asymmetry) as compared to the higher-SES children. In spite of this, perceived stress and boredom related to the task were not related to SES effects. Consequently, this study draws attention to the importance of considering variables related to self-regulation and motivation to control for possible subtle confounds but in the end confirms that the midfrontal mechanisms most responsible for the SES effects on children's selective attention reported here and in previous studies reflect genuine cognitive differences.

### Conflict of interest statement

The authors declare that the research was conducted in the absence of any commercial or financial relationships that could be construed as a potential conflict of interest.

## Appendix A

### Affective questionnaire

#### Pre-testing

**Table:**

What are you feeling right now?
Proud	1	2	3	4	5	I don't understand
	not at all		somewhat		very much	this question
Ashamed	1	2	3	4	5	I don't understand
	not at all		somewhat		very much	this question
Nervous	1	2	3	4	5	I don't understand
	not at all		somewhat		very much	this question
Relaxed	1	2	3	4	5	I don't understand
	not at all		somewhat		very much	this question
Angry	1	2	3	4	5	I don't understand
	not at all		somewhat		very much	this question
Bored	1	2	3	4	5	I don't understand
	not at all		somewhat		very much	this question
Stressed	1	2	3	4	5	I don't understand
	not at all		somewhat		very much	this question
Overwhelmed	1	2	3	4	5	I don't understand
	not at all		somewhat		very much	this question

Proud—You are pleased at yourself, (The parents were proud that their child was a hero).

Ashamed—shame, guilt, disgrace (I was ashamed of my behavior, I know that I should not have acted that way).

Nervous—timid, fearful (I felt nervous when I had to give a speech in front of my school).

Relaxed—become loose, less tense (I always feel relaxed when I am lying on the beach).

Angry—Displeasure (I was very angry when my friend broke my favourite toy).

Stressed—physical or emotional (I feel stressed when I don't have enough time to finish my test).

Overwhelmed—Sometimes if too much is going on all at once, I don't want to be involved anymore, I want to get away or hide in a quiet place.

## Appendix B

### Task appraisal questionnaire

#### Post-testing

**Table:**

1. How did you feel during the task?
Eager	1	2	3	4	5	I don't understand this question
	not at all		Somewhat		very much	
Nervous	1	2	3	4	5	I don't understand this question
	not at all		Somewhat		very much	
Confident	1	2	3	4	5	I don't understand this question
	not at all		Somewhat		very much	
Bored	1	2	3	4	5	I don't understand this question
	not at all		Somewhat		very much	
2. How stressful was the task?	1	2	3	4	5	I don't understand this question
	not at all		Somewhat		very much	
3. How well were you able to do the task?	1	2	3	4	5	I don't understand this question
	not at all		Somewhat		very much	
4. How difficult was the task?	1	2	3	4	5	I don't understand this question
	not at all		Somewhat		very much	
5. How scary/intimidating was the task?	1	2	3	4	5	I don't understand this question
	not at all		Somewhat		very much	
